# On the occurrence of the snakefish *Trachinocephalus myops* (Aulopiformes: Synodontidae) in the Azores archipelago

**DOI:** 10.1111/jfb.70266

**Published:** 2025-11-11

**Authors:** Iryna Hulevata, Filipe M. Porteiro, Eva Giacomello, Diana Catarino

**Affiliations:** ^1^ University of the Azores, Institute of Marine Sciences – OKEANOS Horta Portugal; ^2^ University of Szczecin Szczecin Poland; ^3^ IMAR‐Institute of Marine Research, University of the Azores Horta Portugal

**Keywords:** DNA barcoding, new record, Northeast Atlantic, species distribution

## Abstract

The snakefish *Trachinocephalus myops* is an Atlantic species distributed in tropical and temperate coastal waters on sandy substrates. This study reports the validated record of an adult *T. myops* in the Azores archipelago caught by a fisherman at Faial Island. Meristic, morphometric characters and molecular analyses supported species identity. The possibilities of recurrent misidentification or recent colonization of the species in the region and its biogeographic affinities are discussed. *T. myops* is hereby included as a native species in the Azores.

*Trachinocephalus myops* (Forster, 1801), commonly known as snakefish, is a coastal species within the family Synodontidae Gill, 1861 living in the Atlantic Ocean. *T. myops* was considered a circumtropical monotypic genus; however, recent taxonomic studies split the species from the Indo‐West and Pacific Ocean in various species (Polanco et al., 2016; Prokofiev, 2019). Currently, *T. myops* inhabits only the Atlantic Ocean (Polanco et al., 2016), whereas *Trachinocephalus atrisignis*, *Trachinocephalus trachinus* and *Trachinocephalus gauguini* are new species recognized from the Indic and Pacific Oceans (Polanco et al., 2016; Prokofiev, 2019). The amphiatlantic distribution of *T. myops* spans through the Western Atlantic, from Cape Cod, Massachusetts, to Brazil, including the Bahamas, the Antilles, the Gulf of Mexico and the offshore island of Bermuda, and in the Eastern Atlantic from Mauritania to Gabon, including Cape Verde, Saint Helena and Ascension Island (Hanel & John, 2015; Anderson et al., 1966; Polanco et al., 2016).

*T. myops* occurs in tropical and temperate coastal waters over sandy or muddy bottoms (Simon et al., 2013). It is known for its burrowing behaviour leaving only its eyes exposed. It is an ambush predator, using its sharp teeth and camouflaged bodies to strike at unsuspecting prey (Russell et al., 2020; Sulak, 1990). It primarily feeds on small fish and crustaceans, and it is prey of large fish such as barracudas and groupers (Randall, 1967). *T. myops* inhabits depths ranging from 0 to 400 m, but it is mostly found between 3 and 90 m (Carpenter, 2002; Polanco et al., 2016).

The snakefish has a cylindrical elongated body and a distinct blunt and very short snout. Its typical colouration consists in yellow and bluish stripes intercalated along the trunk, all bordered by narrow brownish lines (Polanco et al., 2016). The species has a large, oblique mouth with fine, pointed teeth arranged in multiple rows, adapted to its predatory lifestyle (Polanco et al., 2016). Adult fish typically grow to around 25 cm, but some can reach 40 cm (Lieske & Myers, 1994). Individuals from an Indo‐Pacific congeneric species live up to 7 years (Sadovy & Cornish, 2000).

The Azores is a Portuguese archipelago in the Northeast Atlantic, located at the Mid‐Atlantic Ridge. The archipelago includes nine volcanic islands, many coastal islets and hundreds of seamounts, some reaching shallow waters (Morato et al., 2008). In the Azores, the family Synodontidae is currently represented by a well‐established population of the Atlantic lizardfish *Synodus saurus* (Linnaeus, 1758) (Porteiro et al., 2010; Santos et al., 1997). Some studies reporting the occurrence of the diamond lizardfish *Synodus synodus* (Linnaeus, 1758) in the region (Carneiro et al., 2019) need further validation. Additionally, there is a single historical record of a *T. myops* larva, caught by a midwater trawl off Faial Island in September 1984 (Carneiro et al., 2014, 2019; Morris, 2025), but until now there were no sightings of fully grown individuals in the region. This may suggest that larvae of this species can reach this region from its western or eastern Atlantic populations. The Azores oceanographic regime is greatly influenced by the Gulf Stream and more directly by the Azores Current (Frazão et al., 2022). The Gulf Stream originates in the Gulf of Mexico, runs along the east coast of the United States and transports warm water to the Northeast Atlantic (Frazão et al., 2022). The Azores Current, a dynamic southern branch of the Gulf Stream flowing eastward at about 35° N, south of the Azores islands, is the subtropical convergence in this region (e.g., Alves et al., 2002; Frazão et al., 2022). Ocean currents are known to promote long‐distance dispersal in several fish species (e.g., Evseenko, 2008).

This work unequivocally reports the first documented record of an adult *T. myops* in the Azores, identified through both morphological and molecular data.

On 21 September 2022 a *T. myops* adult fish was caught in Horta Bay (38.53°–28.62°), around 10 m deep, by a fisherman, using a hand hook‐and‐line gear, and donated to Institute of Marine Sciences – OKEANOS, University of the Azores.

The specimen was measured and photographed, and a sample of muscle tissue was preserved in 96% ethanol for genetic analyses. Morphometric and meristic data were collected following Polanco et al. (2016). Sex and maturity stage were assessed following a macroscopic maturity scale based on Holden and Raitt (1974). The specimen was then fixed in 10% formalin, preserved in 70% in ethanol and deposited at the biological reference collection at OKEANOS (COLETA, 2015) with the catalogue number COLETA‐13453.

Total genomic DNA was extracted using the Mag‐Bind Tissue DNA kit (OMEGA bio‐tek, USA) according to the manufacturer's instructions and using the autonomous extractor KingFisher mL (Thermo – Electron Corporation, USA). The cytochrome *c* oxidase subunit I (COI) region from the mitochondrial DNA was amplified by polymerase chain reaction (PCR) using FishF1 and FishR1 primers (Ward et al., 2005). The thermal cycle parameters started with an initial denaturation of 2 min at 94°C, followed by 35 cycles of denaturation of 30 s at 94°C, annealing for 1 min at 50°C, extension for 1 min 35 s at 72°C, with a final extension of 7 min at 72°C. Template‐free mix controls were added to detect possible DNA contamination, and the integrity of the PCR products was assessed by electrophoresis on a 1% agarose gel. The amplified products were purified using ExoSAP‐IT (USB Corporation, USA) and sequenced for both strands commercially at Stab Vida (https://www.stabvida.com/pt, Lisbon, Portugal).

For comparisons purposes, meristic and morphometrics of four specimens of *S. saurus* from the Azores were taken (COLETA numbers 13454; 13456–13458). The four specimens, and one more specimen (COLETA 13455/GenBank PV544882‐ specimen misplaced at the time), were DNA barcoded following the protocol mentioned above.

Sequenced PCR products were compared with those deposited at BOLD Systems (Ratnasingham & Hebert, 2007). Published sequences from the Atlantic Ocean belonging to *T. myops* were retrieved from the public available database GenBank (Benson et al., 2018) and BOLD Systems and were used to build a phylogenetic tree. Sequences of *T. gauguini* and *T. trachinus* from Indian and Pacific Oceans (accordingly to Polanco et al., 2016) were also included for comparison, as well as four sequences from *S. saurus* available at BOLD Systems from Mediterranean, and five sequences from the Azores, belonging to the specimens measured for comparisons (see accession numbers in Figure 1e). A maximum likelihood tree using MEGA 12 (Kumar et al., 2024) was constructed, using 1000 bootstrap replications and applying the best substitution model (Hasegawa‐Kishino‐Yano model, HKY + I, I = 0,64), as suggested by the Model test implemented in MEGA. Phylogenetic reconstruction was performed by aligning and trimming the dataset to the shortest sequence (577 bp). Mean sequence divergence was calculated based on Tamura‐Nei model (Tamura & Nei, 1993), the best model available suggested by the Model test.

**FIGURE 1:**
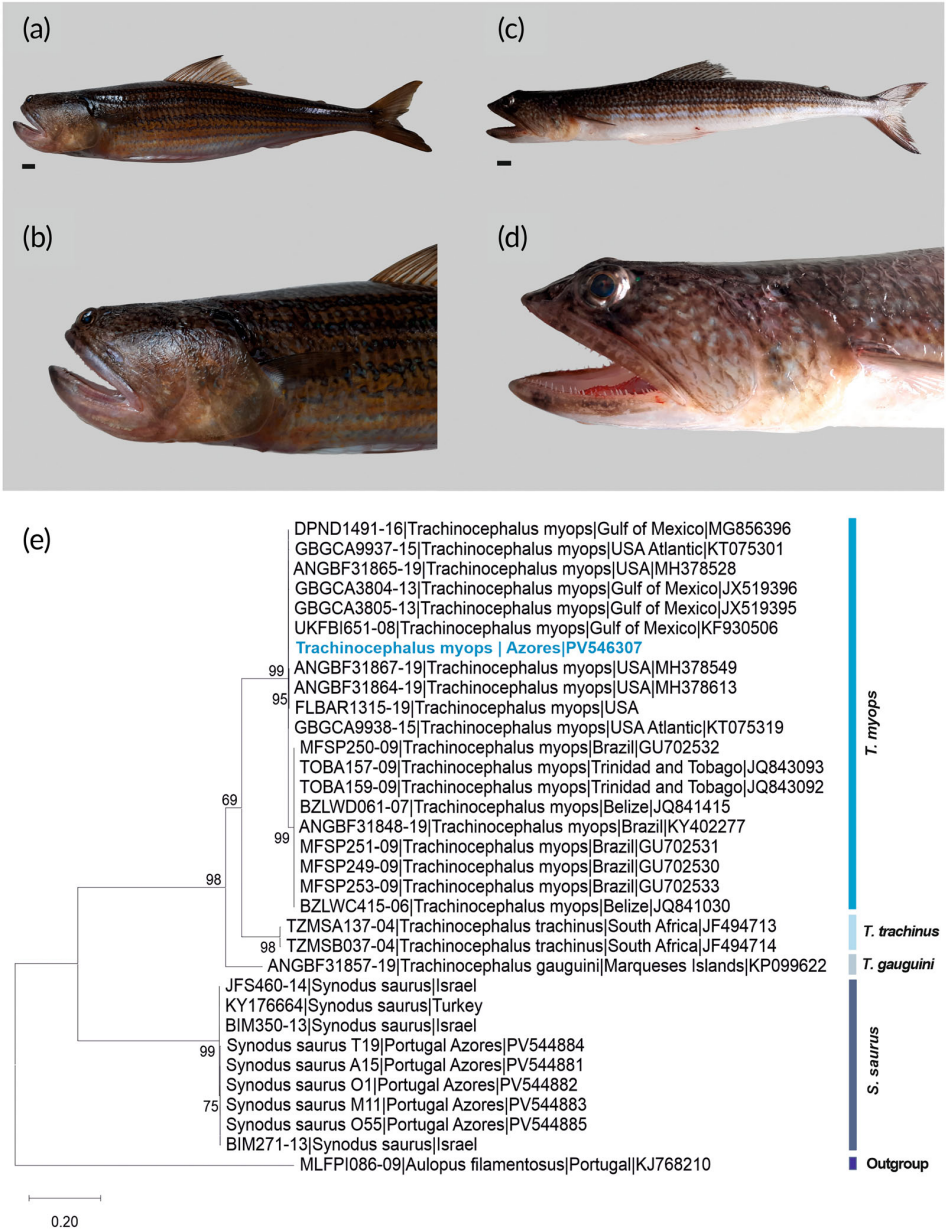
*Trachinocephalus myops* and *Synodus saurus* specimens collected in the Azores archipelago. Photographs of the *T. myops* full specimen while fresh (a), and the head showing a very short snout (b). Photographs of the *S. saurus* full specimen while fresh (specimen PV544885/COLETA 13458) (c), and the head (d). The scale bar in the photographs corresponds to 1 cm. (e) Maximum likelihood phylogenetic tree for *T. myops* and related species obtained using a 577 bp fragment of mtDNA COI. Numbers above internal branches indicate bootstrap values. *T. myops* specimen from the Azores is shown in blue. BOLD Systems and GenBank accession numbers are shown before and after each species name, respectively.

This study did not require specific permits, as the fish was provided by a fisherman after it had died and was not collected specifically for research purposes.

The fresh specimen of *T. myops* had a beige‐brown colouration with dark yellow longitudinal stripes interposed by bluish/greyish stripes, bordered by narrow brownish lines mostly towards the dorsal area (Figure 1a). The specimen had a cylindrical, elongated body with a distinct blunt and very short snout, and a large oblique mouth with fine pointed teeth (Figure 1b). Morphometric measures and meristic counts are presented in Table 1. The specimen was an adult female at spawning stage (with eggs easily observed) measuring 31.7 cm TL (total length). For comparison purposes morphological characters of Azorean *S. saurus* were recorded and are shown in Table 1.

**TABLE 1:** Morphometric measurements (mm), respective body proportions (%) and meristic counts of the *Trachinocephalus myops* and *Synodus saurus* collected in the Azores.

Morphometric characters	*T. myops* Azores		T. myops Polanco et al., 2016	*S. saurus* Azores
	Size (mm)	Body proportion (%)	Body proportion range^a^	Body proportion^a^
Total length (TL)	318			321–378
Furcal length (FL)	290			293–338
Standard length (SL)	265		34.8–242	266–310
Body depth	57.2	21.6	12.8–21.7	10.7–11.6
Body width	43.3	16.3	9.6–19.2	15.0–17.4
Head length	68.9	26.0	24.7–31.3	25.0–26.2
Snout length	9.2	13.4	8.7–14.8	16.4–21.8
Eye diameter	8.6	12.5	10.8–21.7	11.9–15.5
Interorbital width	10	14.5	3.4–11.9	19.7–24.5
Length longest dorsal‐ray	47.4	17.9	14.1–23.9	15.4–16.3
Length longest pectoral‐ray	27.9	10.5	10.5–14.0	10.5–11.6
Length longest pelvic‐ray	55.7	21.0	22.3–30.4	21.0–23.0
Length last dorsal‐ray	20.7	7.8	7.3–11.6	5.8–15.8
Dorsal‐fin base	45.3	17.1	13.8–19.3	14.2–16.9
Anal‐fin base	54.5	20.6	21.1–27.6	12.3–13.5
Distance from snout to origin of pelvic fin	80.1	30.2	30.5–40.0	33.5–36.9
Distance from snout to origin of dorsal fin	107.6	40.6	37.7–45.1	40.8–43.8
Distance from snout to origin of anal fin	183	69.1	62.6–70.5	78.4–81.9
Distance origin dorsal fin to origin of adipose fin	117.9	44.5	40.2–46.7	86.1–88.3

Counts	*T. myops* Azores	Range T. myops Polanco et al., 2016	Range *S. saurus* Azores
Dorsal‐fin rays	13	11–14	12–13
Pectoral‐fin rays	13	11–13	12–14
Pelvic‐fin rays	8		8
Anal‐fin rays	14	13–16	11–13
Predorsal scales	15	15–20	19–20
Lateral line scales	54	53–60	58–59

^a^ Body proportions ranges are in millimetre for TL, FL and SL, and are shown in percentage for the rest of the measurements for *T. myops* from Polanco et al. (2016) and for *S. saurus* from the Azores. Cranial proportions are expressed in percentage of head length (HL), and body proportions are expressed in percentage of standard length (SL). Measurements followed Polanco et al. (2016). Morphometrics and meristics reported for *S. saurus* are based on four individuals.

*S. saurus* showed a greyish‐brown body colouration with a pattern that also resembled beige‐brown stripes along the side of the body intercalated with bluish stripes (Figure 1c). The general body arrangement (shape, body proportions and fins) and colour pattern are somehow similar to that of *T. myops*, with a clear exception of the snout, which is much longer in *S. saurus* (Figure 1b,d).

Amplification of the mtDNA COI barcode sequence from the Azorean *T. myops* specimen resulted in a 636 bp fragment (GenBank accession number: PV546307) >99% similarity to the public reference sequences identified as *T. myops* from USA coast (*n* = 10, accession numbers MH378528, MG856396, JX519395, KF930506, KT075301, JX519396, MH378549, MH378613, KT075319, FLBAR1315‐19) deposited in GenBank and/or at BOLD Systems.

The phylogenetic reconstruction (Figure 1e) shows that the sequence of the Azorean *T. myops* falls into the Atlantic clade, as expected, specifically closely related to *T. myops* from the USA Atlantic coast and northern Gulf of Mexico. The phylogenetic reconstruction suggests the possibility of further separation within Atlantic samples, between North and South America (Figure 1e). The phylogenetic tree also supports genetic separation of *Trachinocephalus* spp. from Atlantic and non‐Atlantic species, and from the out‐group, *S. saurus*. No mtDNA COI sequences of *T. myops* from western Africa are publicly available. The mean sequence divergence between *T. myops* and *T. gauguini* is 17.28 ± 2.05% (mean ± S.E.), 16.46 ± 1.96% with *T. trachinus* and 23.82 ± 2.46% with *S. saurus*, whereas the mean distance between *T. gauguini* and *T. trachinus* is 16.52 ± 1.90%. Sequence divergence between *T. myops* northern and southern Atlantic specimens is 1.67 ± 0.53%.

This study reports the first documented occurrence on an adult fish of *T. myops* in the Azores. Morphometrics and meristic features, the colour pattern, the distinctive eyes’ position and the short and blunt snout positively match the description of adult *T. myops* from other Atlantic populations (Polanco et al., 2016), and molecular data further confirm the identity of the species.

The presence of the species in this region was previously detected by a pelagic offshore larva caught during a mesopelagic survey in the 1980s (MCZ 66756; Carneiro et al., 2014, 2019). The lack of previous references reporting adults of *T. myops* in Azores may be explained by a recurrent misidentification of this species with *S. saurus*, as both species share many morphological and behavioural traits (Sulak, 1990; Soares et al., 2002, 2003; Simon et al., 2013: Russell et al., 2020), although they may be easily distinguished when observed side by side (see Figure 1). As the lizardfishes do not have commercial interest, they are usually discarded at sea immediately after being captured, and therefore the differences between them may have been systematically overlooked by fishers. Moreover, it may be feasible also that researchers studying coastal fishes of the Azores may have overlooked the presence of the species in the region due to the cryptic behaviour of the species and similarities with *S. saurus*. In addition, visual census surveys are often covering areas with rocky or mixed subtract while sandy bottoms are less studied.

Alternatively, to the hypothesis that *T. myops* was disregarded by fishermen and researchers, it is also plausible to assume that *T. myops* might be a newcomer. The recent colonization of Azores waters was reported for many other fish species (e.g., Afonso et al., 2013; Azevedo et al., 2004; Stefanni et al., 2015). However, this raises questions about the source population of such individuals. Do they come from tropical‐subtropical Western Atlantic or from the tropical Western Africa, between Mauritania and Gabon, or Cape Verde islands?

The coastal Azorean ichthyofauna has major biogeographic affinities with Lusitanian Province (Santos et al., 1995; Spalding et al., 2007) and particularly with Madeira and the Canaries archipelagos of the former concept of a wide Macaronesia region (see Freitas et al., 2019). If *T. myops* originated from Western Africa, it would be likely to occur also in Madeira and the Canaries, as seen in other species expanding its geographic distribution due to climate change (Afonso et al., 2013). But contrary to most coastal species that recently colonized the Azores, there are no reported occurrences of *T. myops* in those archipelagos. Moreover, the dispersion of the larvae of *T. myops* from Mauritania or from Cape Verde islands to the Azores would be challenging, considering the actual upper ocean circulation regime in the North Atlantic (i.e., larvae will face the dominant southward flowing Canary Current; Frazão et al., 2022, but see also Santos et al., 1995). In this context, it seems plausible to consider that *T. myops* from the Azores may perhaps originate from the Western Atlantic populations. The prevailing oceanographic regime and currents, such as the tropical‐subtropical North Atlantic Gyre and the eastward‐flowing Azores Current, may be responsible for larval dispersal of *T. myops* from the source population to these islands. However, this hypothesis implies that *T. myops* pelagic larvae and juvenile phases would be long enough to allow for dispersion over such a large distance. The duration of the species pelagic phases is unknown, but the pelagic juveniles can reach more than 4 cm (Anderson et al., 1966), which may be an indicator of a long pelagic life. Although the phylogenetic reconstruction suggests a strong western Atlantic affinity (USA coast and north Gulf of Mexico) for the specimen collected in the Azores (see Figure 1e), samples from the Western Africa populations are currently lacking for further comparisons.

Regardless of the source population of this species in the Azores, the observations reported in this study may illustrate the natural process of colonization (recent or ancient) of remote oceanic archipelagos by coastal benthic organisms. Under certain oceanographic conditions, larvae with specific biological traits disperse regularly through the ocean reaching eventually remote islands. Then, if ecological conditions allow, they settle and thrive as an unpredictable outcome. Despite the volcanic nature of the islands, suitable sandy bottoms are abundant and there are ecological niches available to the settlement of the species in such habitats that show low fish diversity and biomass (Santos & Nash, 1995). The observations of a mature female reported in this study suggest that the species may reproduce in the Azores. However, systematic monitoring will be crucial in determining whether the colonization of these islands by *T. myops* is indeed successful.

The phylogenetic tree shows a separation of the clades of *Trachinocephalus* from the Atlantic and other regions, such as Indian and Pacific oceans, as previously reported (Alavi‐Yeganeh & Bozorgchenani, 2023; Polanco et al., 2016; Susanthi et al., 2023). Although the recognition that the former *T. myops* includes several other species across the globe (Polanco et al., 2016) has been made nearly a decade ago, there is still a lot of confusion and misidentification associated with these species, especially in the online genetic databases, such as GenBank and BOLD Systems. Therefore, we emphasize the need for such databases to be frequently updated to reflect changes in the taxonomic status of the species, to avoid perpetuating such confusions. As also pointed out by Polanco et al. (2016) and Alavi‐Yeganeh and Bozorgchenani (2023), our phylogenetic tree suggested possible further division of *T. myops* within the Atlantic basin, with clade grouping samples from USA coast, northern Gulf of Mexico and Azores, whereas southern Gulf of Mexico and South America were grouped in a distinct clade, with an average sequence divergence 1.67 ± 0.53%. Such results suggest that a more comprehensive phylogeographic and morphological analysis of the Atlantic *Trachinocephalus* should be performed, including samples from Eastern Atlantic, which are currently lacking.

The present findings highlight the importance of integrating morphological and molecular methods for accurate species identification (Bello et al., 2014). Future efforts should include re‐examination of museum specimens and targeted surveys using genetic tools to understand better the true distribution of *T. myops* within the Azorean archipelago and the East Atlantic. The inventory of Azores fish species is hereby updated to include *T. myops* as a dweller species in the Azores.

## AUTHOR CONTRIBUTIONS

Diana Catarino conceived and supervised the study. Diana Catarino and Filipe M. Porteiro sampled the specimens. Eva Giacomello secured funding and managed project. Iryna Hulevata organized and analysed the data, for which the other authors also contributed. Iryna Hulevata and Diana Catarino wrote the first draft of the manuscript, to which all authors contributed.

## FUNDING INFORMATION

This work received national funds through the FCT – Foundation for Science and Technology, I.P., under the project UIDB/05634/2023 and UIDP/05634/2023 and through the Regional Government of the Azores through the project M1.1.A/FUNC.UI&D/003/2021–2024 and the project M1.1.A/REEQ.CIENTÍFICO UI&D/2021/010. Diana Catarino was supported by the national funds through the FCT under the project UIDP/05634/2020 granted to OKEANOS. Eva Giacomello was supported by DEMERSAIS and CONDOR programmes funded by the Azores Government.

## Data Availability

All data are included in the text. Sequences are stored at GenBank, and all accession numbers are provided.
